# Quantifying neutralising antibody responses against SARS-CoV-2 in dried blood spots (DBS) and paired sera

**DOI:** 10.1038/s41598-023-41928-2

**Published:** 2023-09-11

**Authors:** Kelly J. Roper, Jordan Thomas, Wejdan Albalawi, Emily Maddocks, Susan Dobson, Abdullateef Alshehri, Francesco G. Barone, Murielle Baltazar, Malcolm G. Semple, Antonia Ho, Lance Turtle, ISARIC4C Consortium, Cara Donegan, Rebecca G. Spencer, Shona C. Moore, Hayley Hardwick, Tom Solomon, Lance C. W. Turtle, Ana da Silva Filipe, Antonia Ying Wai Ho, Sarah E. McDonald, Massimo Palmarini, David L. Robertson, Janet T. Scott, Emma C. Thomson, Meera Chand, Richard S. Tedder, Nicholas Price, Gary Leeming, Debby Bogaert, Clark D. Russell, Annemarie B. Docherty, Thomas M. Drake, Cameron J. Fairfield, Ewen M. Harrison, Stephen R. Knight, Kenneth A. Mclean, Derek Murphy, Lisa Norman, Riinu Pius, Catherine A. Shaw, Peter W. Horby, Olivia V. Swann, Kanta Chechi, A. A. Roger Thompson, Graham S. Cooke, Shiranee Sriskandan, Charlotte Summers, William Greenhalf, Vanessa Sancho-Shimizu, Saye Khoo, Mahdad Noursadeghi, David Stuart, Lucy Norris, Rishi K. Gupta, Andrew Rambaut, Christoper A. Green, Victoria Shaw, Seán Keating, Gail Carson, Laura Merson, Louise Sigfrid, James Lee, Daniel Plotkin, Marie Connor, Jo Dalton, Chloe Donohue, Carrol Gamble, Michelle Girvan, Sophie Halpin, Janet Harrison, Clare Jackson, Laura Marsh, Stephanie Roberts, Egle Saviciute, Tom Fletcher, Carlo Palmieri, Alison M. Meynert, Murray Wham, Peter J. M. Openshaw, Ryan S. Thwaites, Jake Dunning, Maria Zambon, Gonçalo dos Santos Correia, Matthew R. Lewis, Lynn Maslen, Caroline J. Sands, Panteleimon Takis, Wei Shen Lim, Alexander J. Mentzer, Paul Klenerman, Tassos Grammatikopoulos, Susan Knight, Sarah Tait, J. Kenneth Baillie, Sara Clohisey, Fiona Griffiths, Ross Hendry, Andrew Law, Wilna Oosthuyzen, Beatrice Alex, Benjamin Bach, James Scott-Brown, Petros Andrikopoulos, Marc-Emmanuel Dumas, Julian L. Griffin, Sonia Liggi, Michael Olanipekun, Anthonia Osagie, Zoltan Takats, Wendy S. Barclay, Thushan de Silva, Samreen Ijaz, William A. Paxton, Georgios Pollakis

**Affiliations:** 1 Department of Clinical Infection, Microbiology and Immunology (CIMI), Institute of Infection, Veterinary and Ecological Sciences (IVES), University of Liverpool, Liverpool, L69 7BE UK; 2 Department of Biochemistry and Systems Biology, Institute of Systems, Molecular and Integrative Biology (ISMIB), University of Liverpool, Liverpool, L69 3BX UK; 3 MRC-University of Glasgow Centre for Virus Research, 464 Bearsden Road, Glasgow, G61 1QH UK; 4 Faculty of Health and Life Sciences, Institute of Infection, Veterinary and Ecological Sciences, University of Liverpool, Liverpool, UK; 5 Antimicrobial Resistance and Hospital Acquired Infection Department, Public Health England, London, UK; 6 Blood Borne Virus Unit, Virus Reference Department, National Infection Service, Public Health England, London, UK; 7 Department of Infectious Diseases, Centre for Clinical Infection and Diagnostics Research, School of Immunology and Microbial Sciences, King’s College London, London, UK; 8 Division of Informatics, Imaging and Data Science, Faculty of Biology, Medicine and Health, Centre for Health Informatics, School of Health Sciences, Manchester Academic Health Science Centre, University of Manchester, Manchester, UK; 9 Centre for Medical Informatics, The Usher Institute, University of Edinburgh, Edinburgh, UK; 10 Nuffield Department of Medicine, Centre for Tropical Medicine and Global Health, University of Oxford, Old Road Campus, Roosevelt Drive, Oxford, UK; 11 Department of Child Life and Health, University of Edinburgh, Edinburgh, UK; 12 Department of Epidemiology and Biostatistics, Faculty of Medicine, School of Public Health, Imperial College London, London, UK; 13 Department of Infection, Immunity and Cardiovascular Disease, University of Sheffield, Sheffield, UK; 14 Department of Infectious Disease, Imperial College London, London, UK; 15 Department of Medicine, University of Cambridge, Cambridge, Cambridgeshire UK; 16 Department of Molecular and Clinical Cancer Medicine, University of Liverpool, Liverpool, UK; 17 Department of Pediatrics and Virology, St Mary’s Medical School Bldg, Imperial College London, London, UK; 18 Department of Pharmacology, University of Liverpool, Liverpool, UK; 19 Division of Infection and Immunity, University College London, London, UK; 20 Division of Structural Biology, The Wellcome Centre for Human Genetics, University of Oxford, Headington, Oxford, OX3 7BN UK; 21 EPCC, University of Edinburgh, Edinburgh, UK; 22 Institute for Global Health, University College London, London, UK; 23 Institute of Evolutionary Biology, University of Edinburgh, Edinburgh, UK; 24 Institute of Microbiology and Infection, University of Birmingham, Birmingham, UK; 25 Institute of Translational Medicine, University of Liverpool, Liverpool, Merseyside UK; 26 Intensive Care Unit, Royal Infirmary Edinburgh, Edinburgh, UK; 27 Nuffield Department of Medicine, ISARIC Global Support Centre, Centre for Tropical Medicine and Global Health, University of Oxford, Oxford, UK; 28 ISARIC, Global Support Centre, COVID-19 Clinical Research Resources, Epidemic Diseases Research Group, Oxford (ERGO), University of Oxford, Oxford, UK; 29 Liverpool Clinical Trials Centre, University of Liverpool, Liverpool, UK; 30 Liverpool School of Tropical Medicine, Liverpool, UK; 31 Molecular and Clinical Cancer Medicine, Institute of Systems, Molecular and Integrative Biology, University of Liverpool, Liverpool, UK; 32 MRC Human Genetics Unit, MRC Institute of Genetics and Molecular Medicine, University of Edinburgh, Edinburgh, UK; 33 National Heart and Lung Institute, Imperial College London, London, UK; 34 National Infection Service, Public Health England, London, UK; 35 Department of Metabolism, Digestion and Reproduction, National Phenome Centre, Imperial College London, London, W12 0NN UK; 36 Nottingham University Hospitals NHS Trust, Nottingham, UK; 37 Nuffield Department of Medicine, John Radcliffe Hospital, Oxford, UK; 38 Paediatric Liver, GI & Nutrition Centre and MowatLabs, King’s College Hospital, London, UK; 39 Public Health Scotland, Edinburgh, UK; 40 Roslin Institute, University of Edinburgh, Easter Bush, Edinburgh, EH25 9RG UK; 41 School of Informatics, University of Edinburgh, Edinburgh, UK; 42 Section of Biomolecular Medicine, Division of Systems Medicine, Department of Metabolism, Digestion and Reproduction, Sir Alexander Fleming Building, Exhibition Rd, London, SW7 2AZ UK; 43 Department of Infection, Immunity and Cardiovascular Disease, The Florey Institute for Host-Pathogen Interactions, University of Sheffield, Sheffield, UK; 44 The Roslin Institute, University of Edinburgh, Edinburgh, UK; 45 Virology Reference Department, National Infection Service, Public Health England, Colindale Avenue, London, UK; 46 NIHR Health Protection Research Unit in Emerging and Zoonotic Infections, Liverpool, UK; 47 Respiratory Medicine, Alder Hey Children’s Hospital, Institute in The Park, University of Liverpool, Liverpool, UK; 48 Walton Centre NHS Foundation Trust, Liverpool, UK; 49 Tropical and Infectious Disease Unit, Royal Liverpool University Hospital, Liverpool, UK; 50 Department of Infectious Diseases, Queen Elizabeth University Hospital, Glasgow, UK; 51 NHS Greater Glasgow and Clyde, Glasgow, UK; 52 Transfusion Microbiology, National Health Service Blood and Transplant, London, UK; 53 Department of Infectious Diseases, Guy’s and St Thomas’ NHS Foundation Trust, London, UK; 54 MRC Centre for Molecular Bacteriology and Infection, Imperial College London, London, UK; 55 Clatterbridge Cancer Centre NHS Foundation Trust, Liverpool, L7 8YA UK; 56 Imperial College Healthcare NHS Trust: London, London, UK; 57 Section of Bioanalytical Chemistry, Department of Metabolism, Digestion and Reproduction, Imperial College London, London, SW7 2AZ UK; 58 Department of Microbiology/Infectious Diseases, John Radcliffe Hospital, Oxford University Hospitals NHS Foundation Trust, Oxford, UK; 59 Translational Gastroenterology Unit, Nuffield Department of Medicine, University of Oxford, Oxford, UK; 60 Institute of Liver Studies, King’s College London, London, UK; 61 Section of Genomic and Environmental Medicine, Respiratory Division, National Heart and Lung Institute, Guy Scadding Building, Dovehouse St., London, SW3 3LY UK

**Keywords:** Infectious diseases, Immunology, Microbiology

## Abstract

The ongoing SARS-CoV-2 pandemic was initially managed by non-pharmaceutical interventions such as diagnostic testing, isolation of positive cases, physical distancing and lockdowns. The advent of vaccines has provided crucial protection against SARS-CoV-2. Neutralising antibody (nAb) responses are a key correlate of protection, and therefore measuring nAb responses is essential for monitoring vaccine efficacy. Fingerstick dried blood spots (DBS) are ideal for use in large-scale sero-surveillance because they are inexpensive, offer the option of self-collection and can be transported and stored at ambient temperatures. Such advantages also make DBS appealing to use in resource-limited settings and in potential future pandemics. In this study, nAb responses in sera, venous blood and fingerstick blood stored on filter paper were measured. Samples were collected from SARS-CoV-2 acutely infected individuals, SARS-CoV-2 convalescent individuals and SARS-CoV-2 vaccinated individuals. Good agreement was observed between the nAb responses measured in eluted DBS and paired sera. Stability of nAb responses was also observed in sera stored on filter paper at room temperature for 28 days. Overall, this study provides support for the use of filter paper as a viable sample collection method to study nAb responses.

## Introduction

The severe acute respiratory syndrome coronavirus 2 (SARS-CoV-2) pandemic has been one of the most detrimental viral outbreaks of the twenty-first century and is so far responsible for nearly 6.9 million deaths globally^1^. The rapid development of SARS-CoV-2 vaccines has facilitated large-scale immunisation programmes that have provided vital protection against the virus^2–4^. Measuring antibody responses against SARS-CoV-2 is crucial to understanding population seroprevalence, the longevity of immunity and vaccine efficacy, especially with the emergence of SARS-CoV-2 variants^5^. Humoral immune responses measured from patients infected with SARS-CoV-2 have been found to predominantly target the Spike (S) glycoprotein and Nucleocapsid (N) protein antigens^6^. Many of the antibodies generated against S and N will bind to the viral antigens, but antibodies that can bind and subsequently prevent viral entry, known as neutralising antibodies (nAbs), are the type of antibodies that correlate with protection against future infections^7,8^. Many assays are used to investigate humoral responses against SARS-CoV-2 but not all assays measure nAbs^9^. Live virus assays are the gold standard for measuring nAb responses, but for SARS-CoV-2 they can only be performed in high-containment facilities. Pseudo-virus particle (PVP) assays overcome the containment issues associated with live virus and nAb levels measured against SARS-CoV-2 with PVP assays have been observed to correlate with the results obtained in live virus assays^10–13^.

During the pandemic, traditional serology was limited due to physical distancing restrictions and/or lockdowns imposed by many governments. Dried blood spots (DBS) are an ideal solution to overcome these sample collection issues, as they can be collected from fingerstick blood in non-clinical settings and therefore provide the potential for self-sampling^14^. To date, DBS are most well known for their use in newborn screening for genetic health conditions, but they have also been widely used in diagnosing and monitoring therapy for human immunodeficiency virus (HIV) and hepatitis virus infections^15–17^. The practicality and reliability of DBS to measure antibody responses against a range of viruses has also been demonstrated^17,18^. Many studies have utilised fingerstick DBS (FDBS) to quantify binding antibodies against SARS-CoV-2 S^19–26^ or against both S and N^27–32^. Some studies have also used FDBS to assess SARS-CoV-2 seroprevalence^33–38^, however, only a few studies have explored the use of FDBS to measure SARS-CoV-2 nAb responses^39–41^. We utilised Schleicher & Schuell 903 filter paper cards, previously used to measure HIV viral loads^42^, to store sera, venous blood and fingerstick blood. Samples were utilised from SARS-CoV-2 acutely infected individuals, SARS-CoV-2 convalescent individuals and SARS-CoV-2 vaccinated individuals. By comparing nAb responses in paired sera to eluted filter paper samples we have shown that DBS are a viable sample collection method to study nAb responses against SARS-CoV-2.

## Results

### Human serum retains neutralising ability against SARS-CoV-2 S after storage on filter paper

**Figure 1:**
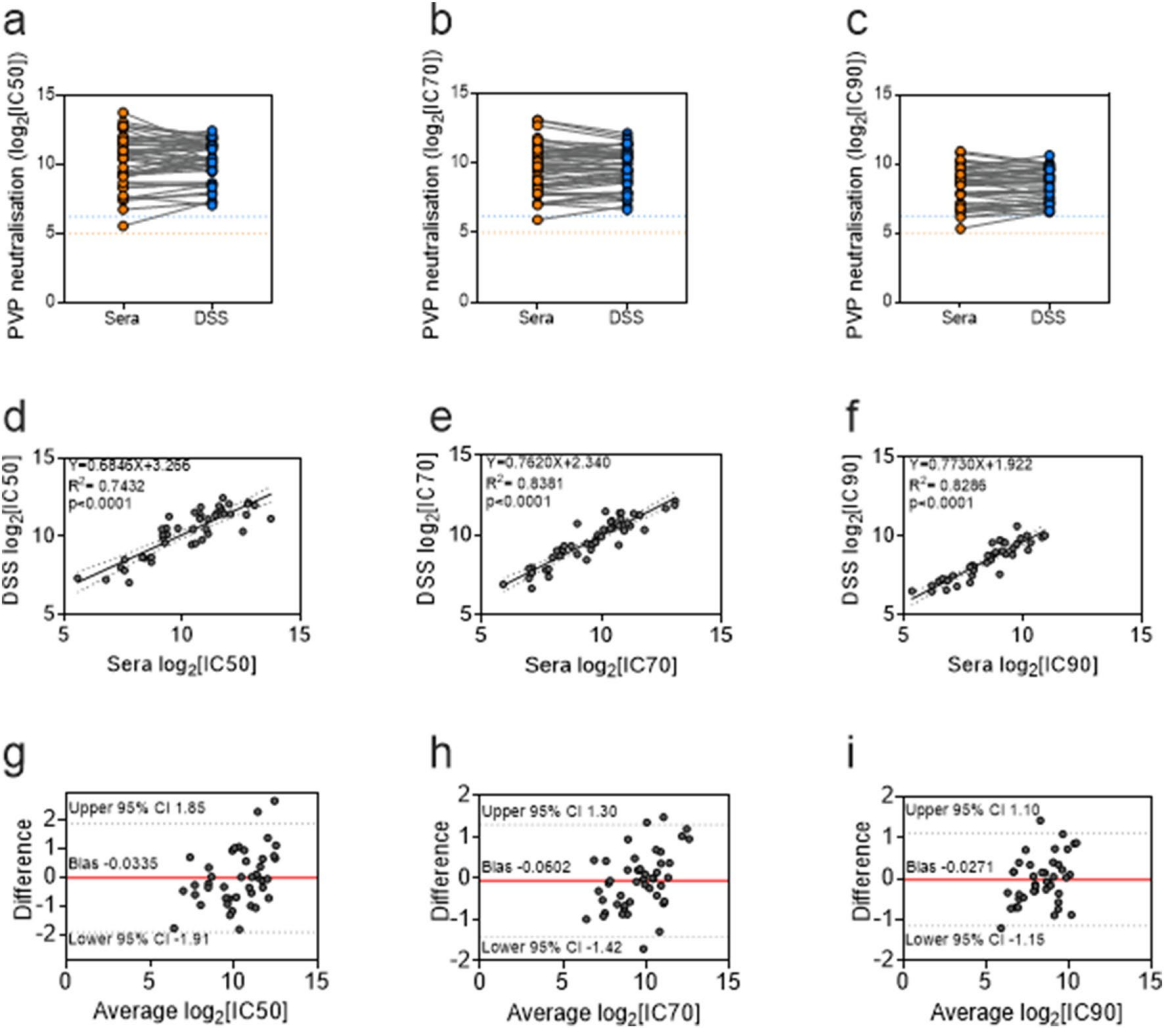
Serum stored on filter paper retains neutralising capacity against SARS-CoV-2 spike. Pseudo-virus particles (PVP) expressing SARS-CoV-2 spike were used to measure the neutralisation capacity of paired human sera stored in direct aliquots and eluted from dried serum spots (DSS) stored on filter paper. Neutralisation activity was defined as the serum dilution that reduced PVP infectivity by 50%, 70% or 90% (IC50, IC70 or IC90, respectively). (**a**–**c**) Slope charts from left to right display on the y-axis PVP neutralisation as (**a**) IC50, (**b**) IC70 and (**c**) IC90. The x-axes show the sample type with sera represented by orange circles and DSS eluates represented by blue circles. IC50, IC70 and IC90 values for paired sample types are connected via grey lines. The dotted lines across the charts represent the lower limits of detection, with the limit for direct sera in orange and DSS in blue (**d**–**f**) Scatter plots from left to right show direct sera IC50, IC70 and IC90 plotted against DSS eluates IC50, IC70 and IC90 values. Simple linear regression analysis were performed and found significant positive relationships between sera aliquots and DSS eluates IC50 values [n = 43, R^2^ = 0.7432, p < 0.0001], IC70 values [n = 47, R^2^ = 0.8381, *p* < 0.0001] and IC90 values [n = 42, R^2^ = 0.8286, *p* < 0.0001]. The dotted lines represent the upper and lower 95% confidence intervals (CI) of the line of best fit. (g–i) Bland–Altman plots display on the x-axes the average IC50 values (n = 43), IC70 values (n = 47) and IC90 values (n = 42) for sera aliquots and DSS eluates and the difference between the two values on the y-axes. Red lines represent bias, and the dotted lines represent the upper and lower 95% CI.

To determine if filter paper can be used to store human sera for serological testing, we tested dried serum spots (DSS) eluates in neutralisation assays using single-round infectious PVP expressing the SARS-CoV-2 S glycoprotein. For all filter paper eluate (FPE) tested in a neutralisation assay, a paired serum sample was also assayed (Supplementary Table S1). Additionally, one or two replicates of a known positive control serum were tested in every assay to measure reliability between assays (Supplementary Fig. S1). Due to availability of sera, 4 different positive control sera were used over the course of the experiments. Initial experiments were performed to test 3 different sample elution conditions. When compared to the neutralisation observed for paired serum, we found that elution at 4 °C overnight was an optimal protocol for eluting DSS from filter paper (Supplementary Fig. S2). Following this, we tested 53 DSS eluates to further evaluate if storage on filter paper affected neutralising capacity when compared to the standard storage method (Fig. 1). Three sera and DSS eluates failed to prevent PVP infectivity and were classed as non-neutralising and subsequently excluded from further analysis, though results agreed. For the remaining 50 paired samples, the inhibitory concentrations (IC) that reduced PVP infectivity by 50% (IC50), 70% (IC70) or 90% (IC90) were calculated. One serum IC50 value was found to be above the limit of detection (LOD) and 2 IC70 and 6 IC90 values were determined to be below the LOD. All IC values that were found to be outside the limits of detection for sera were also outside the limits for FPE and were excluded from further analysis.

43/49 (88%) DSS eluates had comparable IC50 values to paired sera (Fig. 1a). One DSS eluate had an IC50 value that was below the LOD for FPE and 5 had IC50 values that were above the LOD for FPE. There was a positive relationship between the IC50 values of DSS eluates and the IC50 values of paired sera (simple linear regression analysis R^2^ = 0.7432, *p* < 0.0001) (Fig. 1d). 47/48 (98%) IC70 values were measured for DSS eluates, with 1 below the LOD for FPE (Fig. 1b). A positive relationship was also observed between IC70 values for DSS eluates and sera (simple linear regression analysis R^2^ = 0.8381, *p* < 0.0001) (Fig. 1e). Two DSS eluates IC90 values were below the LOD for FPE meaning 42/44 (95%) IC90 values were measured (Fig. 1c). IC90 values were also positively related between assays (simple linear regression analysis R^2^ = 0.8286 *p* < 0.0001) (Fig. 1f). Bland–Altman analyses on the absolute differences between the IC50 values (Fig. 1g), IC70 values (Fig. 1h) and IC90 values (Fig. 1i) for DSS eluates and sera were also performed to assess the agreement between storage methods^43^. Overall, there was high agreement between methods for all IC values measured. The difference between an IC value measured for a DSS eluate and the IC value measured for its paired sera was then calculated for all samples. The differences were then averaged to determine the bias of one assay over or under-estimating IC values compared to the other. The smallest bias of − 0.0271 was observed for IC90 values (95% confidence interval [CI] 1.10 to − 1.15), followed by IC50 values with a bias of − 0.0335 (CI 1.85 to − 1.91). IC70 values had the largest bias of − 0.0602 (CI 1.30 to − 1.41). The small biases observed indicate nAb responses measured for FPE are highly similar to those measured in sera.

**Figure 2:**
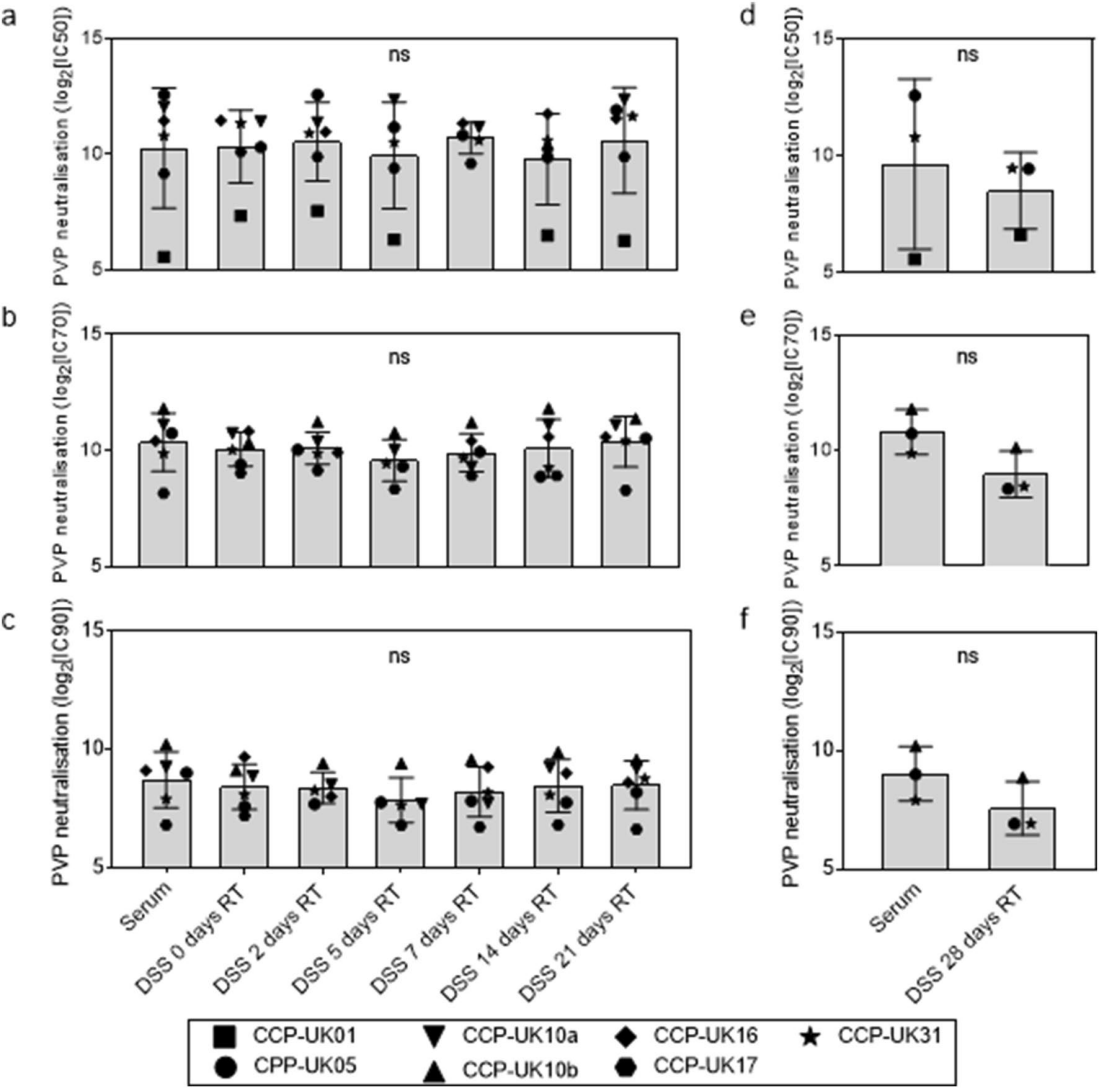
Neutralising capacity of serum against SARS-CoV-2 spike is not significantly affected by storage on filter paper at room temperature for up to 28 days. Single-round infectious pseudo-virus particles (PVP) expressing SARS-CoV-2 spike were used to measure the neutralisation capacity of 7 human sera samples (squares-CCP-UK01, downwards traiangles-CCP-UK10a, upwards triangles CCP-UK10b, circles-CCP-UK05, diamonds-CCP-UK16, hexagons-CCP-UK17 and stars-CCP-UK31). Sera were stored on filter paper kept at room temperature (RT) as dried serum spots (DSS) for 0–28 days before elution and compared to sera stored as direct aliquots at − 80 °C (serum). Due to availability of sera only 4 out of 7 had DSS left at RT for 28 days. Bar charts a-f show neutralisation activity on the y-axes defined as the serum dilution that reduced PVP infectivity by 50% (**a** and **d**), 70% (b and e) and by 90% (**c** and **f**) (IC50, IC70 or IC90, respectively). Error bars represent standard deviation from the mean. Wilcoxon t-tests were run, and no significant differences (ns) were found between the mean IC values for serum controls and the mean IC values for DSS stored at RT from 2 to 28 days (*p* > 0.05).

Next, we tested if PVP neutralisation was affected by storing DSS at room temperature (RT) (approximately 20 °C) over a period of up to 28 days (Supplementary Fig. S3). Seven serum samples were selected and paired DSS were left at RT for 2, 5, 7, 14 and 21 days. Due to limitations in sera availability, only 4 samples (CCP-UK01, CCP-UK05, CCP-UK31, CCP-UK10b) had a DSS left at RT for 28 days (Supplementary Fig. S4). IC70 and IC90 values for CCP-UK01 serum were excluded as they were below the LOD. Overall, 30/39 (77%) IC50 values, 32/33 (97%) IC70 values and 31/33 (94%) IC90 values were measured for eluates from DSS that were stored at RT (Supplementary Table S2). IC50 values for 1 sample (CCP-UK10b) were below the LOD for the FPE for all 6 of the RT DSS eluates tested. One IC90 value (CCP-UK17 eluate from the 2-day RT DSS) was below the LOD due to an elution issue that resulted in a lower eluate volume. A lower volume of eluted sample meant that the detection limit was reduced, though IC50 and IC70 values were still measured. Wilcoxon paired t-tests were performed to assess the difference between the average IC50 values (Fig. 2a and d), IC70 values (Fig. 2b and e) and IC90 values (Fig. 2c and f) for all eluates of DSS stored at RT compared to paired sera and no significant differences (*p* > 0.05) were found. The results indicate that storage of DSS on filter paper cards at RT for up to 28 days did not affect the measurement of nAb responses in FPE.

### Venous whole blood stored on filter paper can act as a surrogate for measuring nAb responses against SARS-CoV-2 S in place of sera

**Figure 3:**
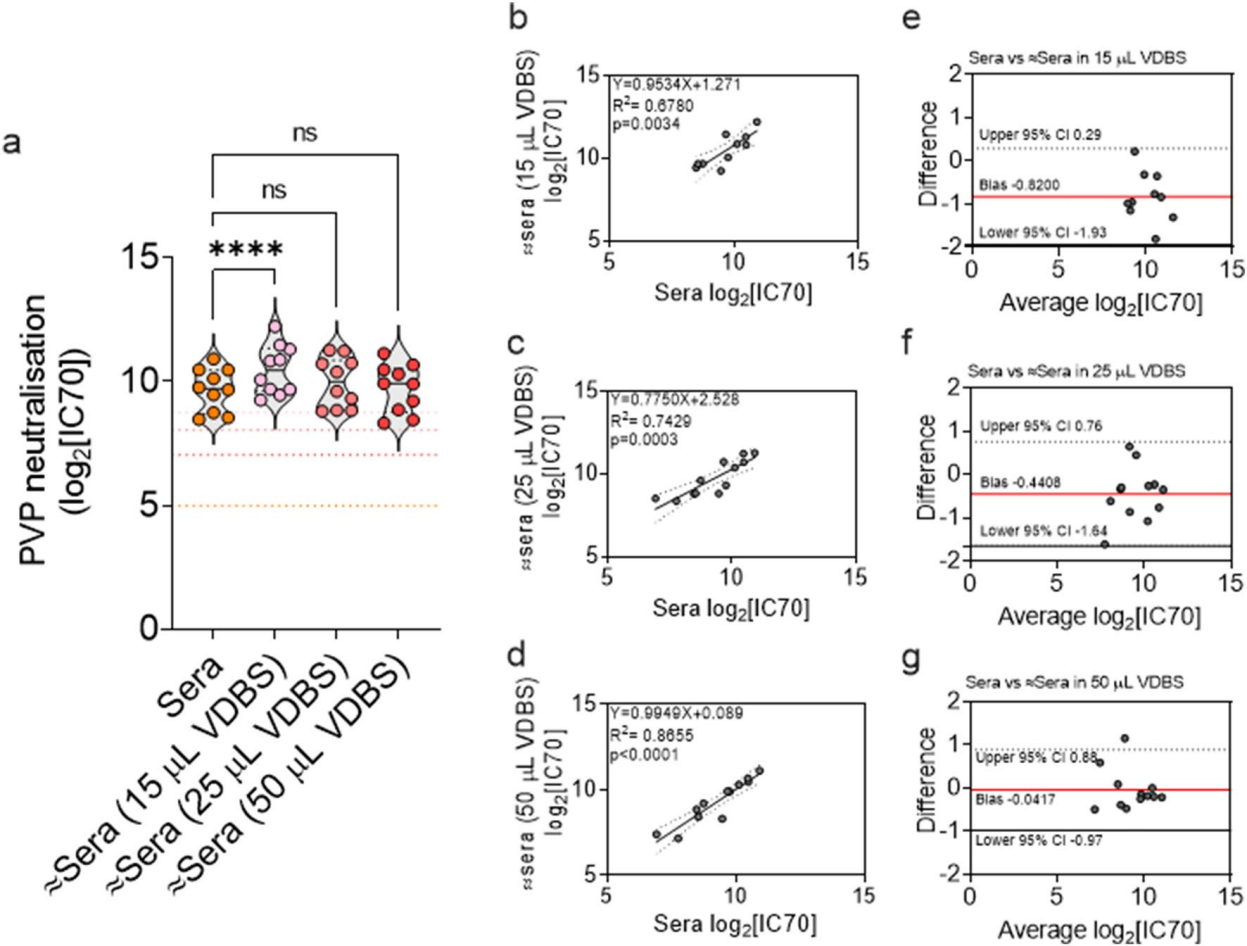
Venous whole blood stored on filter paper shows comparable serum neutralisation of SARS-CoV-2 spike to paired sera aliquots. Pseudo-virus particles (PVP) expressing SARS-CoV-2 spike were used to measure the neutralisation capacity of paired human sera with venous blood eluates that were stored on filter paper as dried blood spots (VDBS). Neutralisation activity was defined as the serum dilution that reduced PVP infectivity by 70% (IC70). As whole blood contains approximately 55% serum this was accounted for when calculating IC70 values for VDBS eluates. (**a**) Violin plot displays on the y-axis PVP neutralisation as IC70 values. The x-axis shows paired sample types (n = 10) with sera represented by orange circles and 3 pipetted volumes of VDBS (15 μL, 25 μL & 50 μL) represented by pink circles. The dotted lines across the plot represent the lower limits of detection, with the limit for sera in orange and VDBS eluates in pink. A repeated measures one-way ANOVA test was run to compare the mean IC70 value for sera against the mean IC70 values for VDBS eluates. A significant difference was found (n = 10, *p* < 0.0001) and a Holm-Šidák’s multiple comparisons test identified it to be between the mean sera and mean 15 μL VDBS eluates IC70 values (*p* < 0.0001). No significant differences (ns) were found between the mean IC70 values for sera and the 25 μL and 50 μL VDBS eluates (*p* > 0.05) (**b**-**d**) Scatter plots show the IC70 values for sera plotted against the IC70 values for VDBS eluates (**b**) 15 μL VDBS (**c**) 25 μL and (**d**) 50 μL VDBS. Simple linear regression analysis found significant relationships between IC70 values for sera and 15 μL VDBS eluates [n = 10, R^2^ = 0.6780, *p* = 0.0034], 25 μL VDBS eluates [n = 12, R^2^ = 0.7429, *p* = 0.0003] and 50 μL VDBS eluates [n = 12, R^2^ = 0.8655, p < 0.0001]. The dotted lines represent the upper and lower 95% confidence intervals (CI) of the line of best fit. (3e–3g.) Bland–Altman plots display on the x-axes the average IC70 values for sera and VDBS eluates (**e**) 15 μL (**f**) 25 μL and (**g**) 50 μL and the difference between the IC70 values on the y-axes. The red lines represent bias, and the dotted lines represent the upper and lower 95% CI.

Following the evaluation of DSS, we proceeded to measure venous DBS (VDBS) eluates in neutralisation assays (Supplementary Fig. S5). Initially 15 μL and 25 μL VDBS eluates along with paired sera were screened for 14 samples and IC70 values were calculated. Two samples were excluded because 1 failed to prevent PVP infectivity and so was classed as non-neutralising and the other had an IC70 value below the LOD. For the remaining 12 samples 50 μL VDBS eluates were also tested. IC70 values were obtained for 12/12 (100%) 25 μL and 50 μL VDBS eluates. Only 10/12 (83%) IC70 values were measured for 15 μL VDBS eluates, with 2 being below the LOD for FPE (Fig. 3). IC70 values for all three volumes of VDBS eluates were found to have a positive relationship with sera IC70 values (Fig. 3b–d). 50 μL VDBS eluates had the strongest relationship with sera (simple linear regression analysis R^2^ = 0.8655., *p* < 0.0001), followed by 25 μL VDBS eluates (simple linear regression analysis R^2^ = 0.7429 *p* = 0.0003) and then by 15 μL VDBS eluates (simple linear regression analysis R^2^ = 0.6780, *p* = 0.0034). Bland–Altman analyses on the absolute differences between the IC70 values for 15 μL VDBS eluates (Fig. 3e), 25 μL VDBS eluates (Fig. 3f) and 50 μL VDBS eluate (Fig. 3g) and sera were also performed, again to assess the agreement of measuring nAb responses between sample types^43^. The smallest bias of − 0.0417 was observed for 50 μL VDBS eluates (CI 0.88 to − 0.97), followed by a bias of − 0.4408 for 25 μL VDBS eluates (CI 0.75 to − 1.64). The 15 μL VDBS eluates had the largest bias of − 0.82 (CI 0.29 to − 1.93). The 10 samples with complete IC70 values for all four sample types (sera, 15 μL, 25 μL and 50 μL VDBS eluates) were analysed in a repeated measures one-way ANOVA test and significant differences (*p* < 0.0001) were found between mean IC70 values (Fig. 3a). A multiple comparisons test was performed to identify which of the mean IC70 values for VDBS eluates were significantly different from the serum controls. The mean IC70 value for 15 μL VDBS eluates was found to be significantly higher (*p* < 0.0001) than the mean IC70 value for the serum controls. No significant differences were identified between the mean IC70 values for the serum controls and the 25 μL and 50 μL VDBS eluates (*p* > 0.05).

### Fingerstick DBS can be used to reliably estimate nAb responses against SARS-CoV-2 S

**Figure 4:**
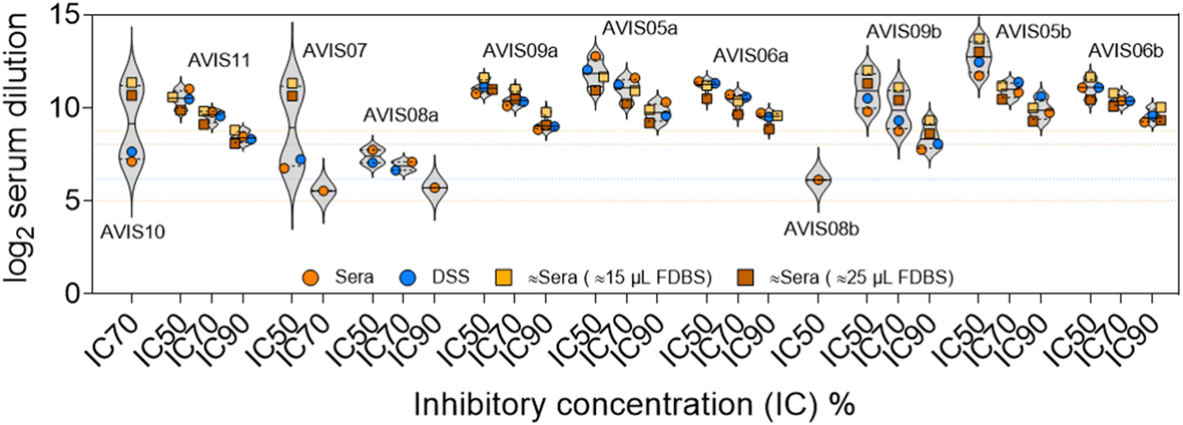
Dried blood spots obtained via fingerstick show comparable serum neutralisation of SARS-CoV-2 spike to paired serum aliquots. Pseudo-virus particles (PVP) expressing SARS-CoV-2 spike were used to measure the neutralisation capacity of human sera and eluted dried blood spots obtained via fingerstick (FDBS) for 11 participant samples. Neutralisation activity was defined as the serum dilution that reduced PVP infectivity by 50%, 70% or 90% (IC50, IC70 or IC90, respectively). As whole blood contains approximately 55% serum this was accounted for when calculating IC values for FDBS eluate. For each FDBS the exact volumes of blood blotted from the participants’ fingers were not measured therefore two volumes were estimated for FDBS volume. The dotted lines across the graph represent the lower limits of detection, with the limit for sera in orange, DSS eluate in blue,  ≈ 15 μL FDBS eluate in yellow and ≈ 25 μL FDBS eluate in brown.

Lastly, we tested 12 FDBS with paired sera to evaluate if capillary blood stored on filter paper could also be used to detect nAb responses against SARS-CoV-2 S PVP (Supplementary Fig. S6). As the exact volume of blood blotted for each FDBS was unknown, known blood spot volumes for VDBS were used to estimate FDBS blood volumes (Supplementary Fig. S7c). From visual comparisons we estimated FDBS to be between 15 and 25 μL of blood. From this estimation, we used two different input volumes of 15 μL and 25 μL to calculate the nAb responses in FDBS. One out of 12 samples tested failed to prevent PVP infectivity and so was classed as non-neutralising and excluded from further analysis. IC50, IC70 and IC90 values were then calculated for the remaining samples (Fig. 4). 1/11 sera IC70 values (AVIS08b) and 3/11 sera IC90 values (AVIS10, AVIS07, and AVIS08b) were below the LOD and were excluded. Following this, 8/11 (73%) IC50 values, 8/10 (80%) IC70 values and 7/8 (88%) IC90 values were measured for both the 15 μL and 25 μL estimated FDBS eluates. For 1 FDBS eluate the IC50 value could not be measured as a result of a poor infectivity curve (AVIS10). Two other IC50 values could not be measured as they were below the LOD for FPE (AVIS08a and AVIS08b) along with 2 IC70 values (AVIS07 and AVIS08a) and 1 IC90 value (AVIS08a). The mean IC50, IC70 and IC90 values that were measured for estimated FDBS eluates and paired sera and were analysed in Friedman ANOVA tests (Supplementary Fig. S8). No significant differences were identified between the mean IC50 values (*p* = 0.079) (Supplementary Fig. S8a) and IC90 values (*p* = 0.052) (Supplementary Fig. S8b). Significant differences were found between the mean IC70 values (*p* = 0.018), but a multiple comparisons test did not find these differences to be between the mean IC70 value for the serum controls and the 15 μL estimated FDBS eluates (*p* = 0.16) or the 25 μL estimated FDBS eluates (*p* = 0.63) (Supplementary Fig. S8c).

Finally, two participants had all filter paper sample types (DSS, VDBS and FDBS) tested in neutralisation assays (Supplementary Fig. S9). Friedman ANOVA tests found no significant differences (*p* > 0.05) between mean IC50 values (Supplementary Fig. S9a), IC70 values (Supplementary Fig. S9b) and IC90 values (Supplementary Fig. S9c) for all sample types tested from the two participants.

## Discussion

The application of FDBS sample collection to study humoral responses could provide a practical alternative to blood collection via traditional venepuncture. FDBS are less invasive, low cost, and can be sampled in the field. This study aimed to assess if storing samples on filter paper affected the quantification of nAb responses against SARS-CoV-2. We observed high levels of agreement between the neutralisation capacity of paired serum samples and FPE, supporting the storage of samples on filter paper to study nAB responses. Other studies have also provided support for the use of DBS to measure SARS-CoV-2 nAb responses^39–41^. Sancilio et al. used a ﻿surrogate virus neutralization test (sVNT) that quantified the inhibition of the interaction between SARS-CoV-2 S and human angiotensin-converting enzyme 2 receptor protein and demonstrated that nAb responses in DBS from SARS-CoV-2 PCR positive and negative samples had a concordance correlation of 0.991 to paired sera^41^. These findings concur with our results, however, PVP assays have been shown in the literature to correlate more closely with live virus assay than sVNTs^12^. Similarly, Danh et al. used a Split-Oligonucleotide Neighbouring Inhibition Assay (SONIA) to measure the ability of nAb to block the spike protein subunit 1-ACE2 interaction^39^. A sensitivity of 91–97% was observed between SONIA and the live virus assay for measuring nAb in sera. SONIA was then used to successfully measure nAb responses in self-collected FDBS. One limitation of measuring responses against the spike protein subunit 1 is that nAbs can be generated against other regions of the spike^44^. Itell et al. also used a PVP assay and found a strong correlation of 0.99 between nAb responses in VDBS eluate and paired sera^40^. However, their results on FDBS were limited, as only two self-collected FDBS were tested with paired sera.

Many studies have found that binding antibodies against SARS-CoV-2 are not affected by storage of DBS at RT for durations ranging from 7 days to 6 months^26,30,40,45,46^. Others have utilised DBS stability for large sero-surveillance studies and have also found antibodies to be stable after transport at ambient temperature^33,34^. No other study, within our knowledge, has measured nAb responses in FPE stored at RT for a defined period. Although nAbs have been shown to be stable in self-collected DBS transported at ambient temperatures, the exact durations that DBS were at RT were not specified^33,40^. We found that DSS eluates retained comparable nAb responses to paired sera after storage on filter paper for 2–28 days at RT. We observed high agreement between nAb responses from FDBS and paired DSS eluates and therefore, we believe that this result, together with previous studies, provides support for the stability of nAb responses against SARS-CoV-2 in DBS stored at RT for up to 28 days. One consideration, however, is that RT varies in different settings and in this UK study, RT was approximately 20 °C. Other countries may experience higher RT conditions. Nevertheless, several studies have stored DBS at temperatures ≥ 37 °C and saw limited effects on measurement of binding antibodies^28,45,46^. The effect of higher temperatures on nAbs would need to be explored further. Overall, it appears the application of DBS sampling within the field may facilitate the removal of high-cost cold-chain handling of samples before analysing a key correlate of immune protection.

Additionally, we demonstrated that VDBS eluate nAb responses strongly agree with nAb responses measured from paired sera. However, we did observe a loss of sensitivity for the smallest volume of VDBS, with the mean IC70 value for the 15 μL VDBS eluate found to be significantly higher than paired sera. The overestimation of nAb responses for 15 μL VDBS eluates was likely due to cutting out excess filter paper area not saturated with blood when VDBS were processed (Supplementary Fig. S7c). As a result, the elution buffer was absorbed into the dry paper surrounding the blood spot and caused the 15 μL VDBS eluates to be more concentrated than estimated. This may explain why the 50 μL VDBS eluate had the highest level of agreement to paired sera as 50 μL of blood was observed to saturate the whole filter paper cut-out. Sensitivity issues connected to blood spot cut-out size have been described previously^14^. One study also noted that blood spot size significantly affected OD values measured^45^. In this study 19 mm sample discs were cut out, but utilising a punch device that cuts out a smaller sample disc size would be one solution to the discussed problem. Another technicality to overcome this issue would be to excise a sub-spot from within the saturated area of the dried sample. This technique has been shown to reliably yield the same volume of serum on average from varying volumes of blotted blood as long as the area within the sub-spot was fully saturated^47^. As smaller volumes of blood are obtained from fingerstick collection, filter paper cut-out size should be considered when using FDBS to improve sensitivity and accuracy.

A limitation of our work was the unknown volume of blood sampled for FDBS. Although we observed that estimated volumes of FDBS were still predictive of nAb responses, we saw decreased sensitivity when measuring IC values (73–88%). The application of quantitative microsampling is one way to overcome the issues associated with unknown sample volume^48^. One example is the neoteryx® Mitra® device, which uses volumetric absorptive microsampling tips to collect either 10, 20, or 30 µL volumes of fingerstick blood. The Mitra® device has successfully been used to measure binding antibodies against SARS-CoV-2 in the field^49,50^. Another type of microsampler used to successfully measure binding antibody responses against SARS-CoV-2 is the Capitainer qDBS, which collects an exact sample volume into a pre-cut DBS disc^51^. However, a limitation of microsampling devices is an increased cost and another simpler solution to measure FDBS volume could be the use of capillary blood tubes (Supplementary Fig. S10).

To conclude, FDBS overcome many of the logistical issues associated with blood collection via standard venepuncture. FDBS are minimally invasive, low cost, stable at ambient temperatures and provide the possibility for self-collection. With growing evidence to support the use of DBS in SARS-CoV-2 serology, the adoption of DBS sampling would greatly benefit large population-based studies and the collection of samples in resource-limited settings. The acceptance of DBS as a viable sample collection mechanism would also aid in future pandemic preparedness.

## Methods

### Ethics and study participants

Participant samples (Supplementary Table S1) used in this work were acquired from two study protocols. Paired serum and DSS were obtained from the Clinical Characterisation Protocol UK (CCP-UK) study which is a part of the International Severe Acute Respiratory and Emerging Infections Consortium (ISARIC) supported by the WHO Ethics Review Committee (RPC571 and RPC572, 25 April 2013). 36 paired samples from both acute (n = 27) and convalescent (n = 9) hospitalised COVID-19 patients were collected between March 2020 and May 2020. Study participants were confirmed to be SARS-CoV-2 positive by reverse transcription polymerase chain reaction (rtPCR) or were highly suspected cases based on clinical presentation. Acute infection samples were collected within 28 days following the onset of symptoms. Convalescent (CV) samples were collected 28 days or more post symptoms onset. Paired serum, DSS, VDBS and FDBS were obtained from the Human Immune Responses to Acute Virus Infections study (AVIS) (Rec reference 16/NW/0160) at The University of Liverpool (protocol number UoL001207). 26 paired samples were collected between June 2020 and June 2022 and were categorized into three sample type groups. The first group were CV samples from recovered participants 28 days or more following SARS-CoV-2 infection (n = 4). The second group were vaccinated samples from participants 28 days or more post-vaccination with either a first, second or third dose of an available SARS-CoV-2 vaccine in the UK (Moderna/Pfizer/AstraZeneca) (n = 7). The samples in the third group were from CV and vaccinated participants who either had a SARS-CoV-2 infection before vaccination, experienced a breakthrough infection following vaccination or experienced both (n = 15). SARS-CoV-2 infection was confirmed by positive rtPCR or by self-reported positive lateral flow test. Informed consent was obtained for all participants across the two study cohorts. Where required all experiments were conducted under accordance with the relevant guidelines and regulations, with experimental protocols approved by the University of Liverpool licensing committees.

### Sample collection

Sera from ISARIC CCP-UK study was received on dry ice in 2 mL screw cap tubes and immediately stored at − 80 °C. For AVIS, sera isolation was achieved by collecting venous blood in silica tubes (BD Vacutainer® SST II Advance Tubes). The venous blood was then left at RT (approximately 20 °C) for a minimum of 30 min before being centrifuged at 1860 g for 10 min to isolate serum. Serum was then aliquoted into 2 mL tubes and stored at − 80 °C until further use. Ahead of use in the neutralisation assay, all sera were thawed and heat-inactivated (HI) at 56 °C for 30 min. HI sera were then centrifuged at 9600 g for 10 min and transferred into new tubes and stored at − 80 °C. Before HI, 50 μL of selected sera were blotted onto Schleicher & Schuell 903 filter paper cards into pre-printed circle guides (Supplementary Fig. S7a) and left to dry for 30 min at RT, before being placed into plastic zip-lock bags and stored at − 80 °C until further use. For a further selected number of samples, DSS cards were left at RT for 2, 5, 7, 14, 21 and 28 days ahead of storage at − 80 °C. VDBS and FDBS were also processed in the same way. For VDBS, 15 μL, 25 μL, 50 μL and 100 μL of venous blood, collected in heparin tubes (BD Vacutainer® Heparin Tubes), were blotted onto Schleicher & Schuell 903 filter paper cards. For FDBS, participant fingers were pierced with a 2.0 mm lancet (UniStik3 extra AT1012) and massaged to encourage blood flow. The resulting blood excretions were then blotted onto Schleicher & Schuell 903 filter paper cards.

### DSS, VDBS and FDBS elution

DSS, VDBS and FDBS were removed from plastic zip-lock storage bags and brought to RT in a microbiology safety cabinet. Dried sample spots were then extracted using a punch device (Supplementary Fig. S7b) that created 19 mm sample discs. The punch device was disinfected with 70% ethanol before and after use. Sample discs were extracted into single wells of a 12-well plate (CytoOne). 300 μL of Phosphate-buffered saline (PBS) buffer was then added to each well containing a sample disc. 1 mL of PBS was added to any empty wells within the plate to minimise evaporation. Plates were then wrapped in parafilm and placed at 4 °C overnight. The next day each sample eluate was collected into a 2 mL tube and HI for 30 min at 56 °C. HI samples were then centrifuged at 9600 g for 10 min to pellet any debris and the resultant supernatant was transferred to a new tube and stored at − 80 °C.

### Cell culture

HEK293T (ATCC® CRL-3216™) cells were cultivated in Dulbecco’s modified eagle medium (Invitrogen) and supplemented with 10% heat-treated FCS (Sigma), 2 mM/mL L-glutamine (Invitrogen), 100 U/mL penicillin (Invitrogen) and 100 mg/mL streptomycin (Invitrogen), termed complete DMEM (Thermofisher). HEK293T/ACE-2 (Creative Biogene CSC-RO0641) cells were used to monitor single-round infectious PVP infectivity and in performing sample neutralisation assays. All cells were cultured at 37 °C and 5% CO_2_.

### PVP production and infection

The ancestral SARS-CoV-2 S glycoprotein (Accession MN908947) was cloned into the pCDNA3.1 expression plasmid (produced by GeneArt Gene Synthesis) and was used in generating PVP stocks via a lentiviral system to generate single-cycle infectious viral particles as previously described^52–54^. HEK293T cells (5.0 × 10^5^ in each well of a 6-well tissue culture flask) (Corning) were grown in 2.0 mL of complete DMEM overnight. Cells were transfected with 750 ng of the lentiviral luciferase reporter construct, pCSFLW, along with 450 ng of the SARS-CoV-2 S expression plasmid and 500 ng of the lentiviral backbone, p8.91, using cationic polymer transfection reagent (Polyethylenimine) (Polysciences) and in the presence of OptiMEM (Invitrogen). OptiMEM/plasmid mix was removed 16 h post-transfection and 2.0 mL complete DMEM was added with the single-cycle infectious SARS-CoV-2 stock harvested 48 h later, passed through a 0.45 µM filter, aliquoted and stored at − 80 °C. PVP infection was monitored on HEK293T/ACE-2 cells by measuring luciferase activity. 100 µL of virus stock was used to infect 1.5 × 10^4^ cells/well for 6 h in a white 96-well plate (Corning). Following infection 100 µL DMEM complete medium was added to each well. 48 h post-infection, media was discarded from the wells and the cells were washed with PBS (Thermofisher), lysed with 30 µL cell lysis buffer (Promega) and luciferase activity determined utilising the commercially available luciferase assay (Promega) and measured using a BMGLabtech FluoroStar Omega luminometer.

### Neutralisation assay

SARS-CoV-2 S enveloped PVP was used in neutralisation assays as previously described^54^. Serum samples from participants were serially diluted twofold with complete DMEM, and 28 µL serum dilution was incubated with 420 µL diluted SARS-CoV-2 PVP for 30 min at RT. 200 µL of virus/serum dilution mix was used to infect HEK293T/ACE-2 cells. The same protocol was followed for dried sample spot eluates (DSS, VDBS and FDBS) with a couple of adaptations. As filter paper eluted samples were already diluted in DPBS following elution from filter paper, only 6 twofold serial dilutions of sample eluates were made in complete DMEM. Then when eluate/DMEM dilution mixes were added to PVP an additional dilution was made by adding 38 µL of neat eluate directly to 420 µL diluted SARS-CoV-2 PVP. Eluate/PVP mixes were then incubated for 30 min at RT before 200 µL of each PVP/eluate mix was used to infect HEK293T/ACE-2 cells. Sera dilutions for DSS, VDBS and FDBS eluates were adjusted accordingly to account for initial dilution in PBS, which was performed to elute the sample from filter paper. As whole blood contains approximately 55% of serum this was also adjusted for when calculating sera dilutions for whole blood samples. Luciferase activity readings of neutralised virus were analysed i) by considering 0% inhibition as the infection values of the virus in the absence of participant sera included in each experiment, ii) by considering 0% inhibition as the infection values of two consecutive high dilutions of participant sera not inhibiting virus entry. The neutralisation activity was defined as the sera dilution that reduced viral infectivity by 50%, 70% or 90% (IC50, IC70 or IC90, respectively).

### Statistical analysis

Statistical analyses were performed using GraphPad Prism 8.0 software. Paired sample comparisons were conducted for all data and individual Figures state the corresponding statistical test performed. The normality of data was assessed by running a D’Agostino and Pearson test. The tests performed include simple linear regression, non-parametric t-tests (Wilcoxon test), repeated measures one-way ANOVA tests and non-parametric Friedman one-way ANOVA tests. Alpha levels of 0.05 were used for all tests and significant *P* values < 0.05 were depicted by *. Bland–Altman plots were also performed to evaluate if dried sample (sera/whole blood) spots eluted from filter paper can be used to measure neutralising antibody responses equivalent to those measured for paired sera.

### Supplementary Information

Supplementary Information.

## Data Availability

The authors welcome requests for access to the data used in this study conducted in response to the COVID-19 outbreak which will be made available by the corresponding author upon reasonable request.
