# Supplementary material for: Quantifying neutralising antibody responses against SARS-CoV-2 in dried blood spots (DBS) and paired sera
Source: Sci Rep. 2023 Sep 11;13:15014. doi: 10.1038/s41598-023-41928-2 (PMC10495436; doi:10.1038/s41598-023-41928-2)
Supplement: Supplementary file 1 — Supplementary Information. [file 41598_2023_41928_MOESM1_ESM.pdf]

Quantifying neutralising antibody responses against SARS-CoV-2 in dried blood spots (DBS) and paired sera.

Kelly J Roper[1], Jordan Thomas[1], Wejdan Albalawi[1], Emily Maddocks[1], Susan Dobson[1], Abdullateef Alshehri[1], Francesco G Barone[2], Murielle Baltazar[1], Malcolm G Semple[1], Antonia Ho[3], Lance Turtle[1], ISARIC4C Investigators[*], William A Paxton[1] and Georgios Pollakis[1,]**

[1]University of Liverpool, Department of Clinical Infection, Microbiology and Immunology (CIMI), Institute of Infection, Veterinary and Ecological Sciences (IVES), Liverpool, L69 7BE, United Kingdom
[2]University of Liverpool, Department of Biochemistry and Systems Biology, Institute of Systems, Molecular & Integrative Biology (ISMIB), Liverpool, L69 3BX, United Kingdom
[3]MRC-University of Glasgow Centre for Virus Research, 464 Bearsden Road, Glasgow, G61 1QH UK.
[*]A list of authors and their affiliations appears at the end of the paper and so are not listed in supplementary material
[**]pollakis@liverpool.ac.uk

1. Supplementary tables

Supplementary Table S1. **Information about serum samples.** Acute samples were taken 0-27 days following symptoms onset from participants hospitalised with COVID-19. Convalescent-hospitalised (CV-h) samples were taken from hospitalised participants who were 28 days post COVID-19 symptom onset. Convalescent (CV) samples were taken 28 days or more following SARS-CoV-2 infection from recovered participants. Vaccinated samples were taken 28 days after vaccination, including after either a first, second or third dose of a SARS-CoV-2 vaccine (Moderna/Pfizer/AstraZeneca). CV-Vaccinated sera were from participants, that either had SARS-CoV-2 infection prior to vaccination, or experienced a breakthrough infection following vaccination, or both. DSS stands for dried serum spots. VDBS stands for venous dried blood spots. FDBS stands for fingerstick dried blood spots. IC values relate to neutralisation activity, defined as the serum dilution that reduced PVP infectivity by 50%, 70% or 90% (IC50, IC70 or IC90, respectively). nn stands for non-neutralising. bld stands for below the limit of detection. ald stands for above the limit of detection. $^{+}$ marks immunocompromised participants. * Mark samples that had IC values determined from multiple replicates of sera (n=6-10). a,b,c respectively correspond to the first, second, third chronological samples from one participant.

Sample ID	Sample date	Sex	Age	Sample classification	Sera dilution for IC50	Sera dilution for IC70	Sera dilution for IC90	Paired samples stored on filter paper
CCP-UK01	19/03/2020	F	58	Acute (13 days)	47	bld	bld	DSS
CCP-UK02	24/03/2020	M	73	Acute (7 days)	nn	nn	nn	DSS
CCP-UK03a	27/03/2020	M	64	Acute (7 days)	609	248	86	DSS
CCP-UK04	27/03/2020	M	56	Acute (7 days)	nn	nn	nn	DSS
CCP-UK05	30/03/2020	F	61	Acute (15 days)	6,090	1,691	512	DSS
CCP-UK06a	30/03/2020	M	72	Acute (24 days)	639	347	130	DSS
CCP-UK07	30/03/2020	F	53	Acute (18 days)	1,337	507	142	DSS
CCP-UK03b	02/04/2020	M	64	Acute (13 days)	334	205	68	DSS
CCP-UK08	07/04/2020	F	52	Acute (17 days)	575	308	96	DSS
CCP-UK09a	07/04/2020	M	58	Acute (9 days)	15,417	8,577	1,132	DSS
CCP-UK06b	07/04/2020	M	72	CV-h (32 days)	6,767	2,355	746	DSS
CCP-UK10a	09/04/2020	F	67	Acute (21 days)	4,217	2,159	614	DSS
CCP-UK11	10/04/2020	M	64	Acute (7 days)	1,777	1,151	414	DSS
CCP-UK12	10/04/2020	M	56	CV-h (28 days)	13,280	6,555	1,920	DSS
CCP-UK13	12/04/2020	M	71	Acute (25 days)	3,503	1,385	396	DSS
CCP-UK09b	14/04/2020	M	58	Acute (16 days)	7,155	2,597	943	DSS
CCP-UK10b	15/04/2020	F	67	Acute (27 days)	13,905	3,552	1,177	DSS
CCP-UK14	16/04/2020	F	65	Acute (15 days)	314	137	40	DSS
CCP-UK15	16/04/2020	M	45	Acute (15 days)	3,013	1,015	365	DSS
CCP-UK16	17/04/2020	M	56	Acute (22 days)	2,770	1,344	547	DSS

CCP-UK17	17/04/2020	F	66	Acute (25 days)	571	282	112	DSS
CCP-UK18	18/04/2020	F	53	Acute (14 days)	168	60	bld	DSS
CCP-UK19	18/04/2020	M	61	Acute (21 days)	ald	8,492	1,760	DSS
CCP-UK20	19/04/2020	M	67	Acute (21 days)	657	336	147	DSS
CCP-UK21	25/04/2020	M	58	Acute (20 days)	1,402	836	398	DSS
CCP-UK22	26/04/2020	F	67	CV-h (40 days)	687	317	107	DSS
CCP-UK23	28/04/2020	M	59	Acute (22 days)	1,510	664	215	DSS
CCP-UK24	29/04/2020	F	52	Acute (19 days)	1853	756	230	DSS
CCP-UK25	02/05/2020	M	57	CV-h (30 days)	1469	509	225	DSS
CCP-UK26	04/05/2020	M	66	CV-h (44 days)	4,035	1,980	551	DSS
CCP-UK27	07/05/2020	F	66	CV-h (38 days)	2,095	672	240	DSS
CCP-UK28	08/05/2020	M	78	Acute (11 days)	8,627	1,799	755	DSS
CCP-UK29	08/05/2020	M	65	CV-h (43 days)	2,180	781	239	DSS
CCP-UK30	10/05/2020	F	18	Acute (10 days)	4,968	1,722	512	DSS
CCP-UK31	13/05/2020	M	57	CV-h (37 days)	1758	920	238	DSS
CCP-UK32	21/05/2020	M	55	CV-h (48 days)	3,189	1,387	422	DSS
AVIS01	18/06/2020	F	44	CV	163*	68*	bld*	n/a
AVIS02	28/08/2020	M	30	CV	91*	49*	bld*	n/a
AVIS03	17/09/2020	F	65	CV	409	223	108	DSS
AVIS04	25/09/2020	F	71	CV	186	127	71	DSS
AVIS05a	26/03/2021	F	59	CV-Vaccinated	6,975	3,105	1,260	DSS, FDBS
AVIS06a	26/03/2021	M	61	CV-Vaccinated	2,728	1,687	841	DSS, FDBS
AVIS07	21/05/2021	F	68	Vaccinated	108	47	bld	DSS, FDBS
AVIS08a	24/05/2021	M	62	Vaccinated	216*	122*	56*	DSS, FDBS
AVIS09a	14/06/2021	F	24	CV-Vaccinated	1,714	1,087	456	DSS, FDBS
AVIS05b	24/06/2021	F	59	CV-Vaccinated	3,383	1,813	852	DSS, FDBS
AVIS06b	24/06/2021	M	61	CV-Vaccinated	2,181	1,320	599	DSS, FDBS
AVIS10	15/07/2021	M	28	Vaccinated	561	138	bld	DSS, FDBS
AVIS08b	26/08/2021	M	62	Vaccinated	70	bld	bld	DSS, VDBS, FDBS
AVIS11	26/08/2021	M	35	CV-Vaccinated	1267*	671*	328*	DSS, VDBS, FDBS
AVIS12[+]	26/08/2021	M	56	Vaccinated	nn	nn	nn	DSS, VDBS, FDBS
AVIS09b	27/10/2021	F	24	CV-Vaccinated	891	427	215	DSS, VDBS, FDBS
AVIS08c	18/05/2022	M	63	CV-Vaccinated	2198	1427	586	VDBS
AVIS13	27/05/2022	M	44	CV-Vaccinated	1691	709	256	VDBS
AVIS14	31/05/2022	M	37	Vaccinated	581	354	199	VDBS
AVIS15	31/05/2022	M	68	CV-Vaccinated	1919	1111	645	VDBS
AVIS16	31/05/2022	F	61	CV-Vaccinated	3753	1944	995	VDBS
AVIS17	01/06/2022	M	61	CV-Vaccinated	474	373	236	VDBS
AVIS18	01/06/2022	F	26	CV-Vaccinated	410	121	60	VDBS
AVIS19	01/06/2022	F	59	Vaccinated	344	217	101	VDBS
AVIS20	07/06/2022	F	52	CV-Vaccinated	2048	1420	713	VDBS
AVIS21	07/06/2022	F	51	CV-Vaccinated	1241	816	386	VDBS

Supplementary Table S2. Dried serum spot (DSS) storage at room temperature (RT). Number of inhibitory concentration (IC) values measured for reduction in SARS-CoV-2 S pseudo-virus particle (PVP) infectivity by 50% (IC50), 70% (IC70) or 90% (IC90) by eluates from DSS that were stored at RT a period of up to 28 days prior to elution.

	**IC50**	**IC70**	**IC90**
**DSS 2-days RT**	6/7	6/6	5/6
**DSS 5-days RT**	5/7	5/6	5/6
**DSS 7-days RT**	5/7	6/6	6/6
**DSS 14-days RT**	5/7	6/6	6/6
**DSS 21-days RT**	6/7	6/6	6/6
**DSS 28-days RT**	3/4	3/3	3/3
**Total:**	**30/39**	**32/33**	**31/33**

2. Supplementary figures

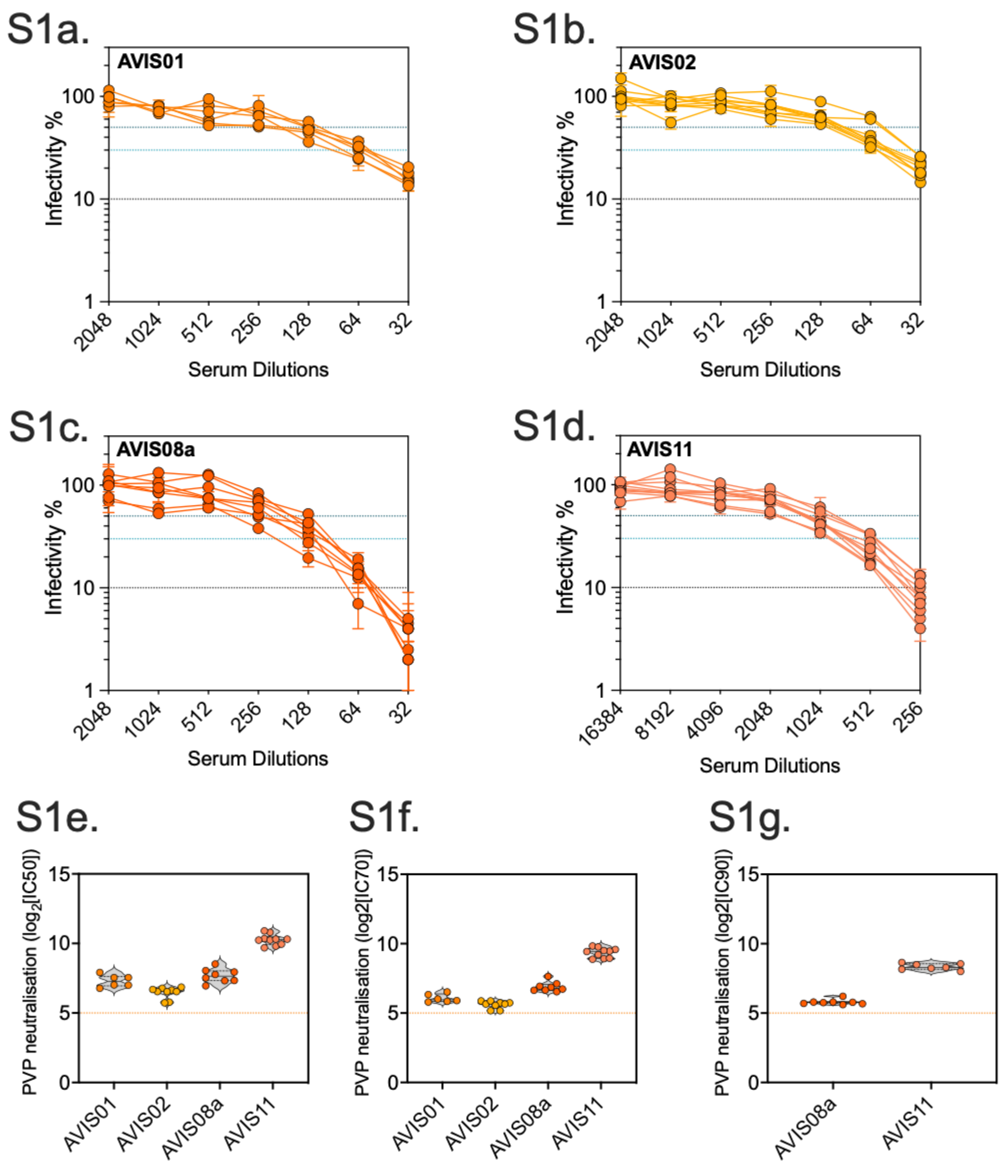

Supplementary Figure S1. Repeatability of positive control sera. The neutralisation capacities of 4 control sera that were repeatedly measured against pseudo-virus particles (PVP) expressing SARS-CoV-2 spike from 4 participants (AVIS01 n=6, AVIS02 n=9, AVIS08a n=8 and AVIS11 n=10). (S1a-d) Infection curves for replicate sera from each of the participants, showing percentage of PVP infectivity plotted against serum dilution. Error bars represent standard error of the mean (SEM) between duplicate technical replicates. The x-axes display the serum dilutions and y-axes the percentage of PVP infectivity. Dotted lines plotted on all graphs mark when 10% infectivity, 30% infectivity and 50% infectivity of PVP were recovered. (S1e-g) Violin plots display the neutralisation activity, defined as the serum dilution that reduced PVP infectivity by 50%, 70% or 90% (IC50, IC70 or IC90, respectively) for each of the 4 participants. The orange dotted line across each of the graphs represents the lower limit of detection for the assay. IC90 values could not be determined for participants AVIS01 and AVIS02 as they were below the limit of detection for the assay.

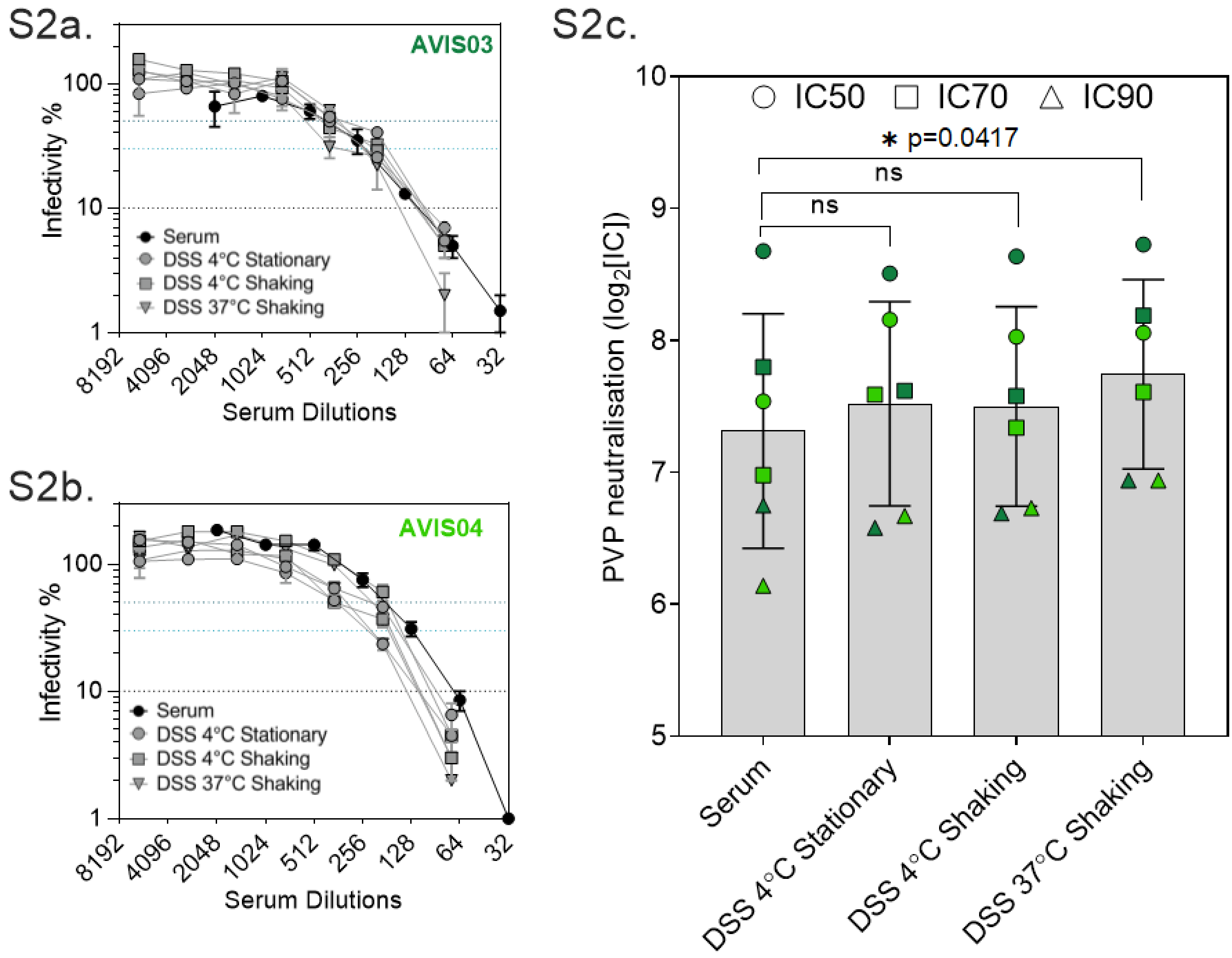

Supplementary Figure S2. Evaluation of optimal protocol for elution of sera from filter paper. Single-round infectious pseudo-virus particles (PVP) expressing SARS-CoV-2 spike were used to measure the neutralisation capacity of paired human sera stored in direct aliquots and on filter paper as dried serum spots (DSS). Three different elution conditions were trialled to extract DSS from filter paper, with 300 µL of PBS added to each 50 µL DSS with subsequent incubation overnight at either 4°C stationary, 4°C shaking or 37°C shaking. Sera tested were from two participants (AVIS03 and AVIS04). Duplicate DSS from each donor were tested in all conditions. (S2a) AVIS03 (S2b) AVIS04 (S2a and S2b) PVP infection curves for each of the DSS conditions are shown (grey circles 4 °C stationary, grey squares 4 °C shaking and downward triangles 37 °C shaking) along with a paired serum sample (black circle). Error bars represent standard error of the mean (SEM) between duplicate technical replicates. The x-axes display the serum dilutions and y-axes the percentage of PVP infectivity. Dotted lines plotted on all graphs mark when 10% infectivity, 30% infectivity and 50% infectivity of PVP were recovered. (S2c) Bar chart shows neutralisation activity on the y-axis defined as the serum dilution that reduced PVP infectivity by 50% (circles), 70% (squares) or 90% (upwards triangles) (IC50, IC70 or IC90, respectively). Error bars represent standard deviation from the mean. IC values quantified for samples from participant AVIS03 are represented by dark green symbols and by light green circles AVIS04. The different DSS elution conditions are shown along the x-axis. For DSS the mean IC value read from duplicate DSS curves for each elution condition are plotted. A Friedman ANOVA test found a significant difference between the average PVP neutralisation of DSS samples and the serum control (p=0.292). A Dunn's multiple comparisons test identified the significant difference to be between the serum control and the DSS eluted at 37 °C shaking (p=0.0417). No significant differences (ns) were identified between the serum and the DSS conditions at 4 °C (p>0.05).

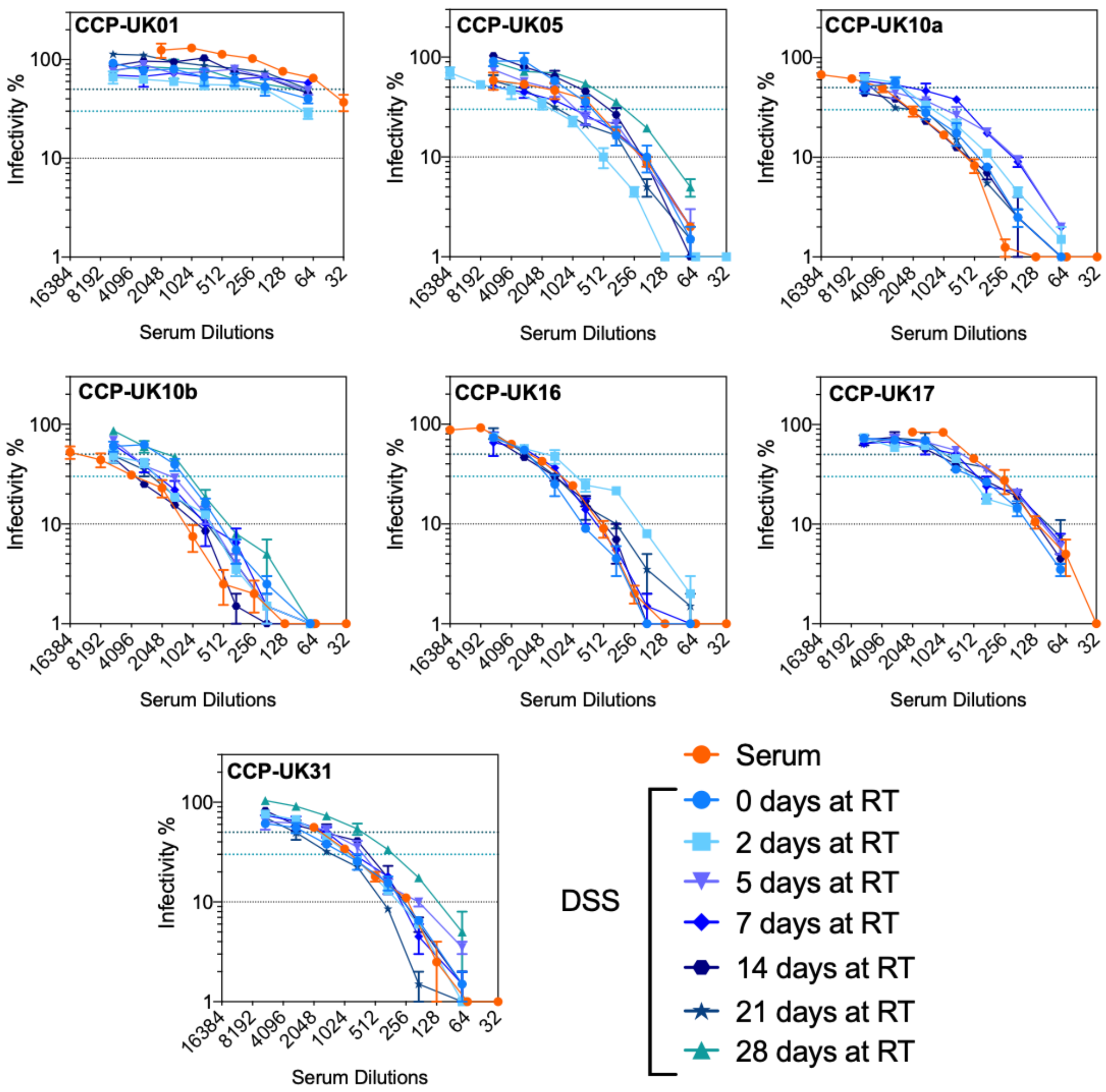

Supplementary Figure S3. Serum stored at room temperature for up to 28 days on filter paper retains neutralising capacity against SARS-CoV-2 spike. Single-round infectious pseudo-virus particles (PVP) expressing SARS-CoV-2 spike were used to measure the neutralisation capacity of 7 human sera samples (CCP-UK01, CCP-UK05, CCP-UK10a, CCP-UK10b, CCP-UK16, CCP-UK17 and CCP-UK31). Sera were stored on filter paper kept at room temperature (RT) as dried serum spots (DSS) for 0-28 days and compared to sera stored as direct aliquots at -80 °C (serum). Due to availability of sera only 4 out of the 7 sera had DSS left at RT for 28 days. The infection of PVP at different serum dilutions is displayed by a curve for each sample type. Error bars represent standard error of the mean (SEM) between duplicate technical replicates. The x-axes display the serum dilutions and y-axes the percentage of PVP infectivity. Dotted lines plotted on all graphs mark when 10% infectivity, 30% infectivity and 50% infectivity of PVP were recovered. Serum samples are represented by orange circles, DSS 0 days at RT by blue circles, DSS 2 days at RT by blue squares, DSS 5 days at RT by blue downward facing triangles, DSS 7 days at RT by blue diamonds, DSS 21 days at RT by blue stars and DSS 28 days at RT by blue upward facing triangles.

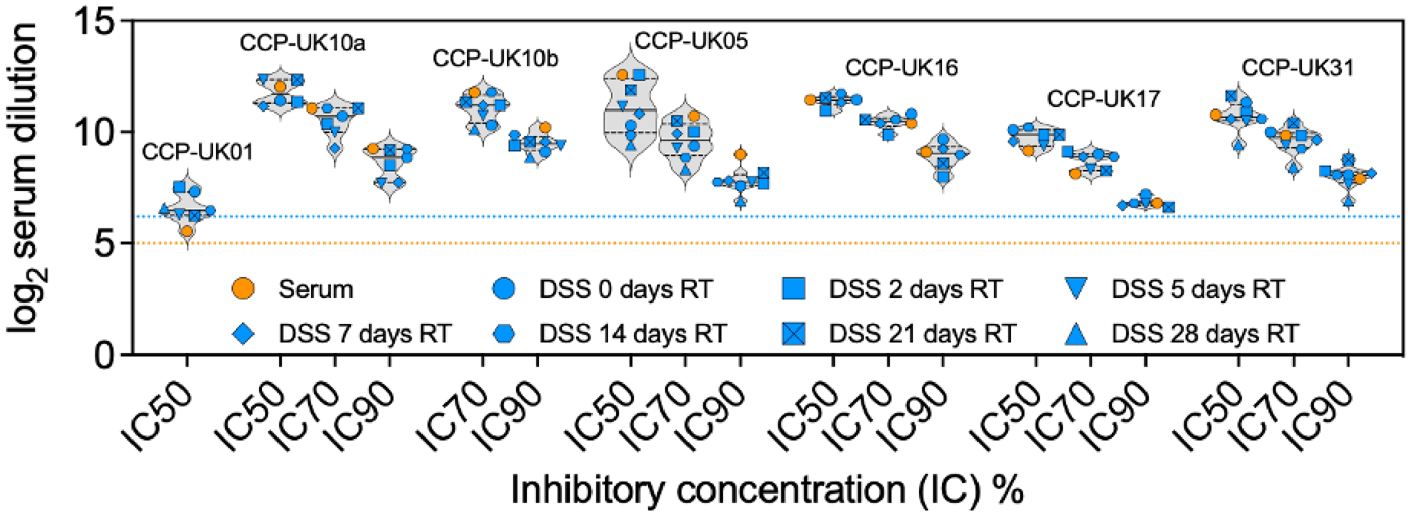

Supplementary Figure S4. Sera stored at room temperature for up to 28 days on filter paper retains neutralising capacity against SARS-CoV-2 spike. Pseudo-virus particles (PVP) expressing SARS-CoV-2 spike were used to measure the neutralisation capacity of 7 human sera (CCP-UK01, CCP-UK10a, CCP-UK10b, CCP-UK05, CCP-UK16, CCP-UK17 & CCP-UK31). Dried serum spots (DSS) were stored on filter paper and kept at room temperature (RT) for 0-28 days before elution and compared to sera stored in direct aliquots at -80 °C (serum). Due to the availability of sera, only 4 out of the 7 samples had paired DSS left at RT for 28 days. Neutralisation activity was defined as the serum dilution that reduced PVP infectivity by 50%, 70% or 90% (IC50, IC70 or IC90, respectively). The dotted lines across the graph represent the lower limits of detection, with the limit for direct sera in orange and DSS eluate in blue.

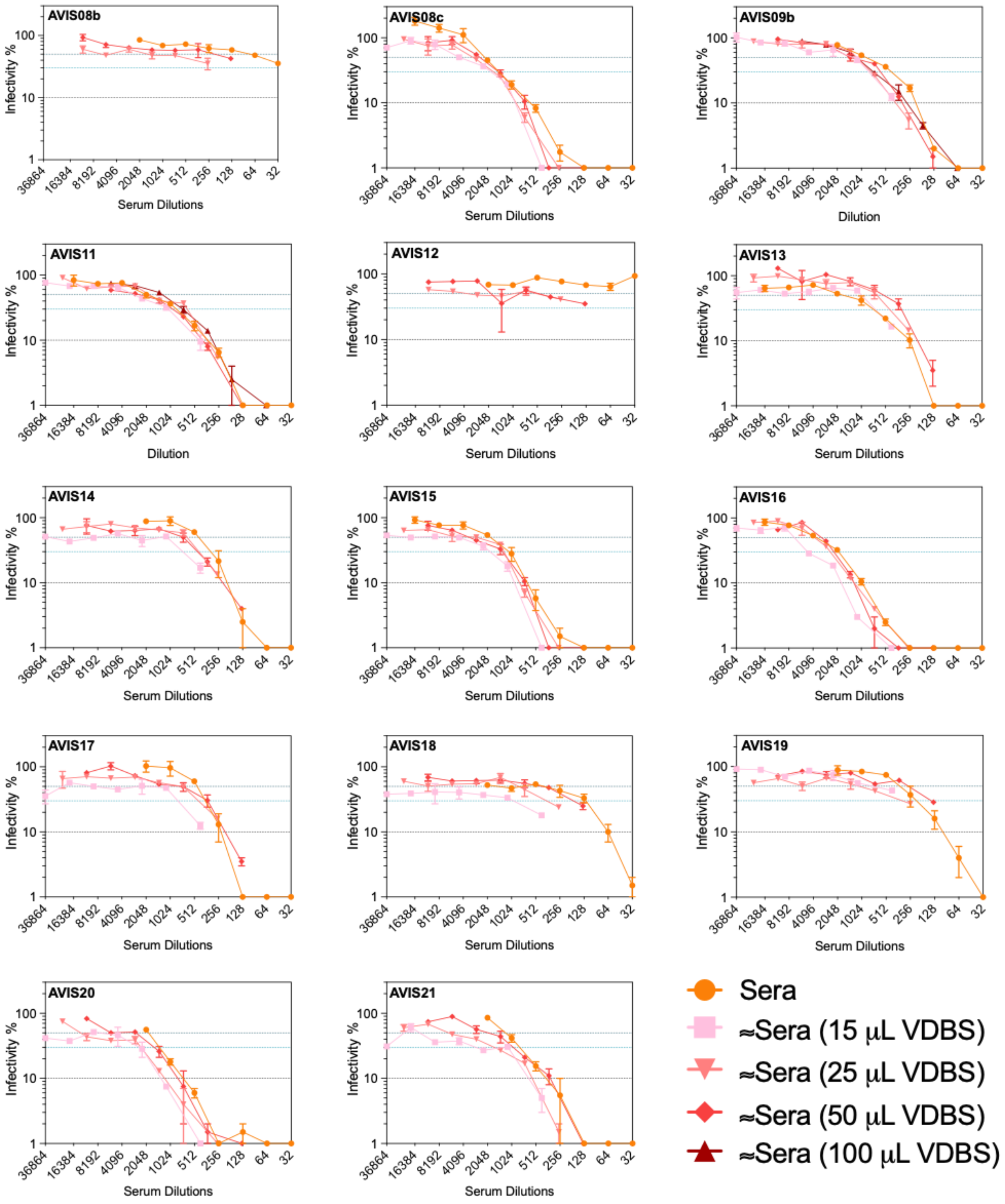

Supplementary Figure S5. Neutralisation curves of SARS-CoV-2 spike pseudo-virus particles (PVP) for paired human sera and venous whole blood stored on filter paper. Single-round infectious PVP expressing SARS-CoV-2 spike were used to measure the neutralisation capacity of 14 paired human sera and two-four pipetted volumes of venous dried blood spots (VDBS) eluates. The percentage of PVP infectivity achieved at serial dilutions of sera were plotted for all sample types. Error bars represent standard error of the mean (SEM) between duplicate technical replicates. Serum samples are represented by orange circles, 15 μL VDBS by pink squares, 25 μL VDBS pink downwards triangles, 50 μL VDBS by a pink/red diamonds and 100 μL VDBS red upwards facing triangles. Dotted lines plotted on all graphs mark when 10% infectivity, 30% infectivity and 50% infectivity of PVP were recovered. The sample ID is displayed in the top left corner of each graph.

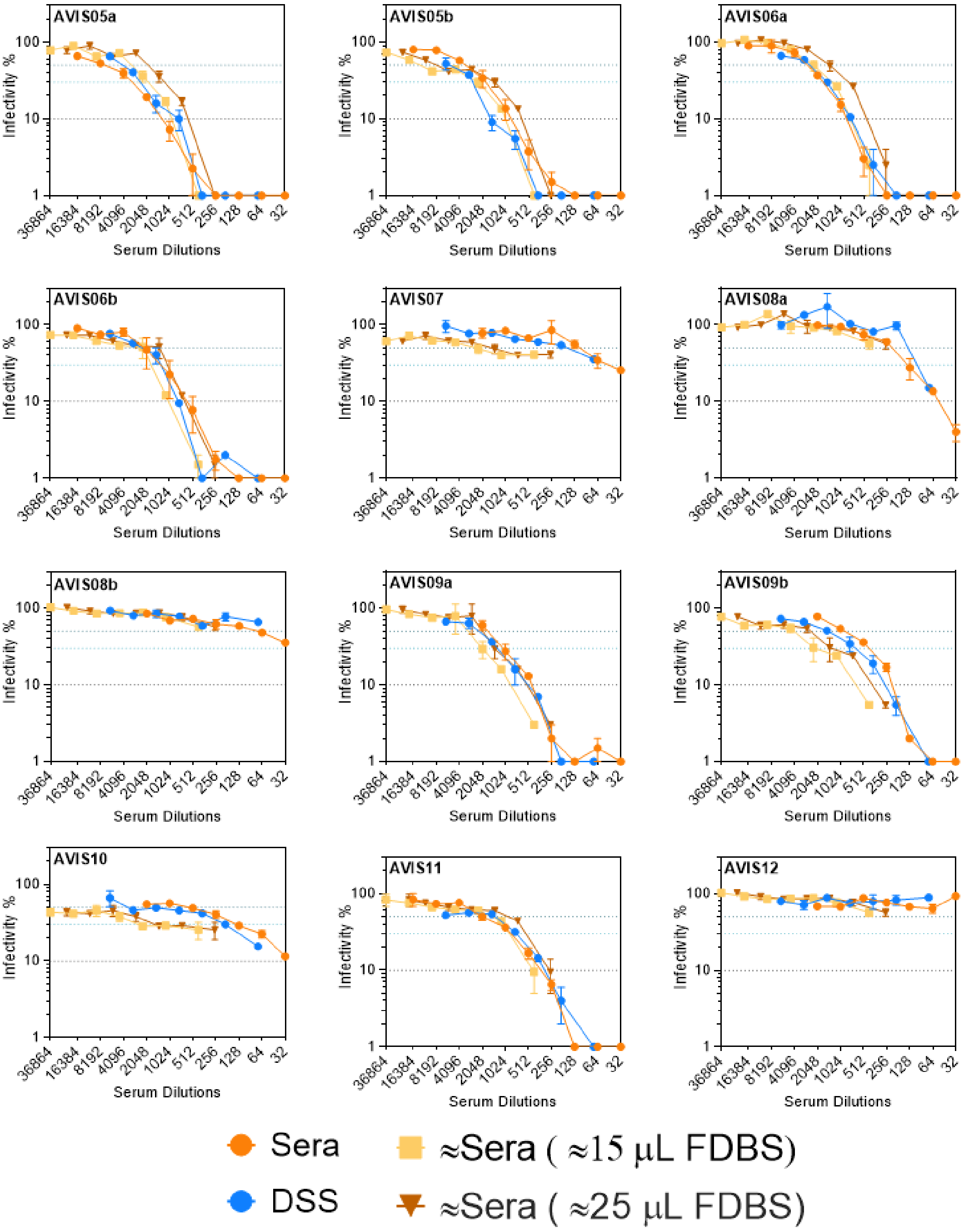

Supplementary Figure S6. Neutralisation curves against SARS-CoV-2 spike for paired human sera and fingerstick dried blood spots (FDBS). Single-round infectious pseudo-virus particles (PVP) expressing SARS-CoV-2 spike were used to measure the neutralisation capacity of 12 paired human sera, dried sera spots (DSS) eluates and FDBS eluates. The percentage of PVP infectivity achieved at serial dilutions of serum were plotted for all sample types. Error bars represent standard error of the mean (SEM) between duplicate technical replicates. Sera aliquots are represented by orange circles and DSS eluates by blue circles. For FDBS eluates the exact volume of blood blotted from the participant's finger was not measured therefore two volumes of blood were estimated, 15 µL FDBS estimates are represented by yellow squares and 25 µL FDBS estimates by brown squares. Dotted lines plotted on all graphs mark when 10% infectivity, 30% infectivity and 50% infectivity of PVP were recovered. The sample ID is displayed in the top left corner of each graph.

S7a.

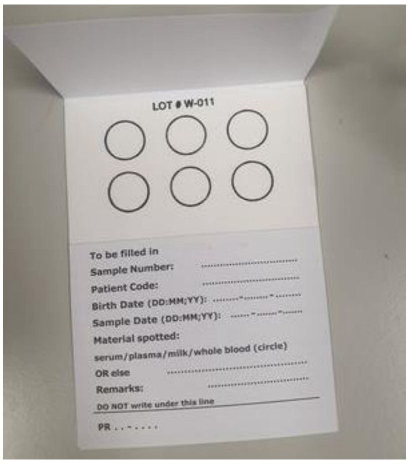

S7b.

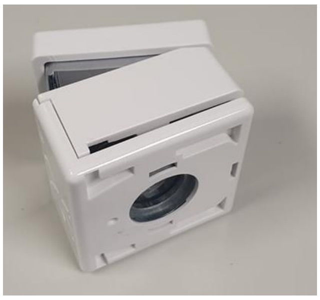

S7c.

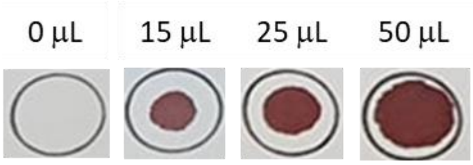

Supplementary Figure S7. Images of filter paper (Schleicher & Schuell 903) sampling process. (S7a) Filter paper card used for sample collection. (S7b) Punch device used to cut out 19 mm sample discs from filter paper. (S7c) Images of 15 µL, 25 µL and 50 µL dried whole blood spots stored on filter paper.

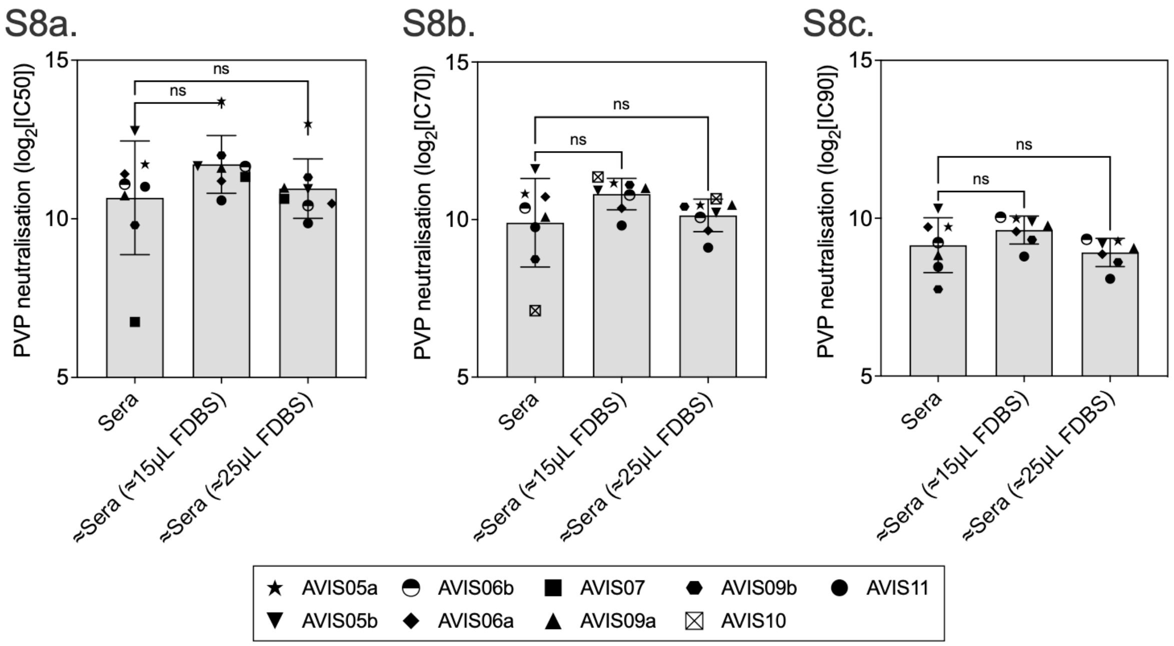

Supplementary Figure S8. **Dried blood spots obtained via fingerstick sampling can be used to assess neutralising antibody response against SARS-CoV-2 spike comparable to sera aliquots.** Single-round infectious pseudo-virus particles (PVP) expressing SARS-CoV-2 spike were used to measure the neutralisation capacity of 9 paired human sera and fingerstick dried blood spots (FDBS). Each paired sample is represented by the same symbol across all bars. For the FDBS eluate the exact volumes of blood blotted from participants' fingers were not measured therefore two volumes were estimated for the FDBS volume (15 µL and 25 µL). (S8a-c.) Bar charts show neutralisation activity on the y-axes defined as the sample dilution that reduced PVP infectivity by (S9a) 50%, (S9b) 70% or (S9c) 90% (IC50, IC70 or IC90, respectively). As whole blood contains approximately 55% serum this was accounted for when calculating IC values for FDBS eluates. Error bars represent standard deviation from the mean. Friedman ANOVA tests were run, and no significant differences (ns) were found between the mean IC50 and IC90 values for serum and FDBS eluates ($p>0.05$). Significant differences were found between the mean IC70 values ($p=0.0179$), but a follow up Dunn's multiple comparison test found no significant differences between the mean FDBS eluate IC70 values and the serum control IC70 value ($p>0.05$).

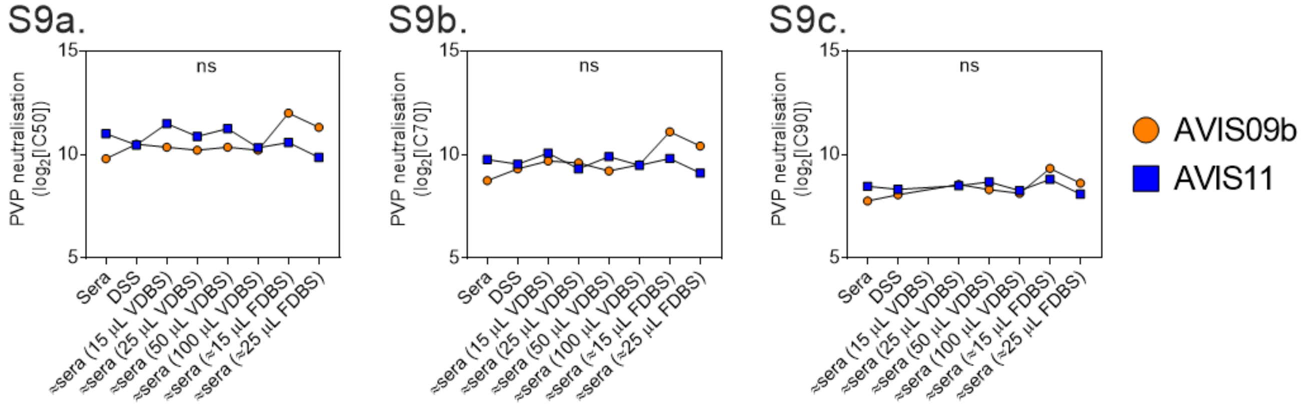

Supplementary Figure S9. Samples stored on filter paper retain neutralising capacity against SARS-CoV-2 spike comparable to paired sera aliquots. Pseudo-virus particles (PVP) expressing SARS-CoV-2 spike were used to measure the neutralisation capacity of paired human sera, dried serum spot (DSS) eluates, dried venous blood spot (VDBS) eluates, and fingerstick dried blood spot (FDBS) eluates. For FDBS the exact volumes of blood blotted from the participants' fingers were not measured therefore two volumes were estimated for FDBS volume (15 µL or 25 µL). Neutralisation activity was defined as the serum dilution that inhibited PVP infectivity by 50%, 70% or 90% (IC50, IC70 or IC90, respectively). As whole blood contains approximately 55% serum this was accounted for when calculating IC values for DBS eluate. Line graphs display on the y-axes PVP neutralisation as (S9a) IC50, (S9b) IC70 and (S9c) IC90. The x-axes show the sample type. Friedman ANOVA tests were run, and no significant (ns) differences were found between any of the mean IC values for each sample type when compared to the mean IC value for control sera ($p>0.05$).

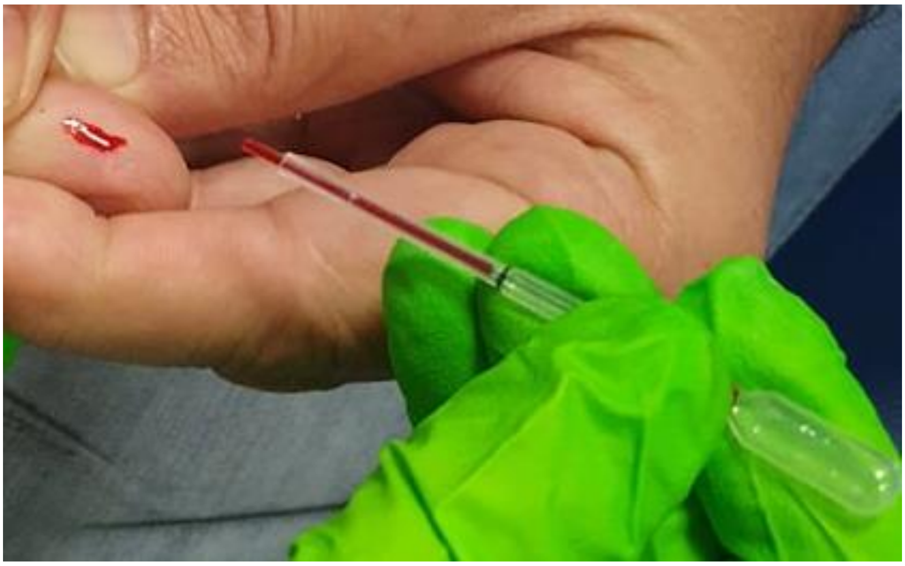

Supplementary Figure S10. **Fingerstick blood collection using capillary tube**. Collecting 40 µL of blood in capillary tube (ptscollect™ 40 µL; product code 2866). Finger pierced with 2.00 mm lancet (UniStik3 extra, product code AT1012).
